# The School Nutrition and Meal Cost Study-I: Overview of Findings Related to Improving Diet Quality, Weight, and Disparities in US Children and Policy Implications

**DOI:** 10.3390/nu13041357

**Published:** 2021-04-19

**Authors:** Mary Story, Lindsey Miller, Megan Lott

**Affiliations:** Healthy Eating Research, Duke Global Health Institute, Duke University, 310 Trent Drive, Duke Box 90519, Durham, NC 27708, USA; lindsey.miller102@duke.edu (L.M.); megan.lott@duke.edu (M.L.)

The national school breakfast and lunch programs administered by the United States Department of Agriculture (USDA) are a cornerstone of the nation’s nutrition safety net for children from low-income families. The National School Lunch Program (NSLP) makes it possible for all school children in the United States to receive a nutritious lunch every school day. Almost all public schools in the U.S. (95%) participate in the program, providing lunches to more than 30 million children on an average day; of these 21.5 million are children from low-income families. In 2019, 14.7 million children from low-income families participated in the national School Breakfast Program (SBP); 80 percent were free and another 5 percent were provided at a reduced price.

The School Nutrition and Meal Cost Study (SNMCS), a nationally representative study conducted in 2014−2015 to assess school meal programs and school food environments, was funded by USDA with a contract to Mathematica (Mary Kay Fox, Principal Investigator) to lead the study including design, methods, data collection, analysis and results. The study addressed (1) school meal program operations and school nutrition environments; (2) food and nutrient content of school meals and afterschool snacks and overall nutritional quality of meals; (3) school meal costs and school foodservice revenues; and (4) student participation, student and parent satisfaction, plate waste, and students’ dietary intakes. The SNMCS provides crucial information about the nutritional quality and costs of producing school meals after implementation of the federal Healthy, Hunger Free Kids Act (HHFKA) of 2010. This landmark legislation resulted in transformative reforms to the school lunch and breakfast programs for the first time in over 30 years. As a result, USDA updated nutrition standards for school breakfast and lunch for the first time in 15 years; established nutrition standards, titled Smart Snacks, for items sold outside of the reimbursable school meal programs, and updated requirements for local school wellness policies. Many of these changes went into effect between 2012 and 2014. Thus, the SNMCS was able to evaluate the impact of the updated nutrition standards.

The SNMCS data was collected from nationally representative samples of school food authorities (SFAs), schools participating in the NSLP, and students attending the schools. The study collected data from 518 school food authorities (SFAs), over 1200 schools, 2165 students, and 1850 parents; and also included plate waste observations for 6253 lunch trays and 3601 breakfast trays. The data collection involved self-administered web-based surveys of SFA directors, school nutrition managers, and school principals; a web-based menu survey, competitive foods checklists, cafeteria environment observations, plate waste observations, 24-h dietary recalls of students, measurement of students’ height and weight, student and parent surveys, meal cost interviews with SFA and school staff, and collection of administrative cost data. The SNMCS offers the most comprehensive and methodologically robust data on school meals available to date. Released in April 2019, the SNMCS produced four volumes of reports summarizing study findings which are available online (https://www.fns.usda.gov/school-nutrition-and-meal-cost-study, accessed on 11 January 2021). 

Healthy Eating Research, a national program of the Robert Wood Johnson Foundation (RWJF) based at Duke University, received funding from RWJF to support a series of original studies analyzing data from the SNMCS to gain insight on the impact of the HHFKA on the nutritional quality of meals served and sold in school, students’ weight status, and to identify ethnic/racial, income, and geographic disparities that may exist. The articles included in this Special issue seek to identify how we can further improve school meal programs and school food environments and identify promising strategies related to obesity prevention. The *Nutrients* Special Issue includes 15 papers.

The key research questions answered in this Special Issue were identified as research priorities by an advisory committee of experts in nutrition and school food policy. The lead authors developed consistent methods across analyses, including variable definitions. The core themes explored in this Special issue include: (1) the nutritional quality of meals served and sold in schools; (2) student diet quality and weight status; and (3) the role of state and local policies, and universal free meals. The key findings described below offer evidence needed by advocates, and policy-makers to inform and guide critical policy action and dialogue to improve school food environments and programs at the local, state, and federal levels.

1. The Nutritional Quality of Meals Served and Sold in Schools. The SNMCS documented that the nutritional quality of meals served and sold in schools has significantly improved since implementation of the updated federal nutrition standards. 

Bardin and colleagues [1] found no significant disparities in the nutritional quality of NSLP lunches across race/ethnicity and poverty subgroups. Students in higher poverty schools, and those with majority Black or majority Hispanic students had less access to competitive foods (i.e., foods and beverages sold outside of school meals) than students in higher income and majority White schools. Higher poverty schools and majority Black and Hispanic schools were more likely to have a school and district wellness policy. Overall, results suggest that high poverty and majority Black and Hispanic schools have more healthful school food environments than other school types.

Fox and colleagues [2] evaluated the levels of added sugars in school meals and children’s dietary intakes. The majority of schools exceeded the Dietary Guidelines for Americans limit for added sugars (no more than 10% of calories from added sugars daily) at breakfast (92%), while 69% exceeded the limit at lunch. The leading source of added sugars was flavored milk, followed by sweetened cold cereals and condiments/toppings at breakfast, and flavored milk and cake at lunch. On average, school breakfasts and lunches provided 163 calories/day from added sugars (88 calories at breakfast and 75 calories at lunch). 

Cohen and colleagues [3] found that HEI scores for competitive entrees were an average of 30 points lower than HEI scores for lunches, with greater differences in small and/or rural schools compared with large and urban schools. Furthermore, 99% of commonly served potential competitive food entrees (e.g., pizza, hotdogs, chicken nuggets) did not meet Smart Snack nutrition standards, primarily due to higher sodium and saturated fat levels. 

Chriqui and colleagues [4] found on average, most beverages sold in middle schools (84.5%) and high schools (74%) were compliant with the Smart Snack beverage standards; it is encouraging that so many schools were compliant during the first year of implementation. 

Policy Implications. Findings from these studies demonstrate that children in the US, regardless of where their school is located, have access to healthy school breakfasts and lunches. It is evident that the nutritional quality of school meals has improved since updated nutrition standards were implemented in 2012. Yet, ongoing attempts to roll back the nutrition standards could significantly jeopardize the healthfulness of foods and beverages available to students. Study findings support that sales of competitive food entrees should continue to be limited, or be required to meet Smart Snack nutrition standards, as these items tend to be less healthful options in comparison to the reimbursable school meal. Evidence on added sugars and flavored milk expose a significant gap in the current nutrition standards and given the high added sugar consumption documented by school-age children, demonstrate the urgency for establishing an added sugar maximum limit for school meals. Further, given the large contribution of flavored milk to added sugar intakes in school meals, USDA should restrict or limit flavored milk at school.

2. Student Diet Quality and Weight Status. School meals contribute more than one-third, and up to half, of the students’ total daily calorie intake. Given the high prevalence of child obesity among school-age children, it is important to provide healthy meals at school and to continue to assess the impact of the updated nutrition standards on student diet quality and weight status. 

Bardin et al. [5] explored the relationship between student weight status and participation in NSLP and SBP after implementation of the HHFKA (2012–2013) and found no clear association between usual participation (three or more days a week) in the NSLP or SBP and student weight status suggesting that school breakfasts and lunches do not lead to higher BMIs.

Schwartz and colleagues [6] found that stronger state nutrition policies for competitive foods and beverages were associated with lower student BMI scores. 

Chriqui et al. [4] found that in schools offering more beverages meeting Smart Snack standards, students were less likely to consume unhealthy drinks during the school day.

Gearan et al. [7] found that lunches consumed by NSLP participants had significantly higher total HEI scores (indicating higher nutritional quality) than lunches consumed by nonparticipants in both lower-income and higher-income subgroups and White and Black students. The nutritional quality of school lunches had a positive influence on students’ overall diet (over a 24-h period). 

Forrestal et al. [8] affirmed that students in food-insecure households were more likely to participate in NSLP than food-secure students. School meals contributed significantly more calories to food-insecure students’ diets than to food-secure students. For all students, dietary intakes from school meals were of higher dietary quality than foods eaten the rest of the day (nonschool foods). These findings indicate that USDA school meals promote diet quality among all students, but are especially important contributors to the diets of children in food-insecure households.

Policy Implications. Implementation of the updated HHFKA school nutrition standards has increased the healthfulness of school breakfast and lunches and resulted in a positive impact on children’s diets. Children who participate in the NSLP or SBP receive better nutrition quality via school meals than nonparticipants who may be eating from vending machines, purchasing a la carte items, or bringing foods from home. Schools meals were also found to be of higher quality than foods eaten outside of school and can be healthier than foods eaten the rest of the day. While USDA school meals promote dietary quality for all students, they are especially important for children living in food-insecure households, indicating the importance of safeguarding and preserving the NSLP and SBP nutrition standards, making the programs more accessible to lower income households, and reducing stigma from participation.

Importantly, there is no evidence that school meals are contributing to children gaining excess weight. Further, implementing strong federal and state competitive food and beverage standards is associated with lower student BMIs, likely due to reduced availability of unhealthy foods and beverages in schools. Policy makers should prioritize protecting the Smart Snack standards and states can institute stronger policies to further limit the availability of unhealthy foods and beverages in schools. 

3. The Role of State and Local Policies, and Universal Free Meals. The HHFKA renewed and expanded the requirement that US school districts participating in federal child nutrition programs must develop and implement a local wellness policy (LWP). LWPs differ across the country as districts are allowed flexibility in policy development. Two papers also examined Universal Free Meals. 

Long and colleagues [9] evaluated whether meal costs varied by Universal Free Meal (UFM) status and found that participation was associated with lower per meal costs in the breakfast program and marginally lower costs in the lunch program among medium and large schools (over 500 students). HEI scores did not vary significantly by UFM status. This suggests that UFMs can provide nutritious meals to more students without a financial disadvantage for schools and school districts. 

Cohen and colleagues [10] conducted a systematic review to examine universal free school meals and students’ school meal participation and diet quality, academic performance, and BMI. Nearly all studies found positive associations with universal free meals and participation, most studies found a positive association with diet quality, food security, and academic performance. Several studies also found that universal free meals may have a protective effect on BMI. 

Leider et al. [11] found that strong district policies were associated with more students eating and liking school breakfast. Students in schools that served breakfast free of charge to all students were more likely to eat the school breakfast. This is important since student participation in the SBP is generally low.

Schwartz et al. [6] found that stronger state nutrition policies for competitive foods were associated with higher odds of having fewer or no unhealthy competitive foods and beverages available. 

Piekarz-Porter et al. [12] found that school district wellness policies with nutrition standards for what can be sold during the school day were more likely to have a corresponding healthy district food procurement policy. Districts were more likely to have procurement policies on saturated fats and added sugars when mentioned in the wellness policy. 

McLoughlin and colleagues [13] found that practices such as providing nutrition information for menu items, promoting vegetables, involving students and parents in menu planning were positively associated with strong district or state laws and policies. 

Turner et al. [14] found that having a strong policy requiring evaluation was associated with implementation of local wellness policies, and that having definitive provisions in policies was associated with implementation and use of these practices.

Chriqui and colleagues [15] found that states with wellness policy requirement laws reported implementing more nutrition-related practices. State wellness policy requirement laws were associated with district local wellness policies comprehensiveness and district-level implementation. 

Policy Implications. These results suggest that strong state and local policies that go beyond the federal standards and are implemented as intended can increase student access to healthier foods and beverages, increase NSLP and SBP participation, and reduce costs. The findings related to universal free meals shed light on a policy opportunity to increase participation in school meals, while reducing costs and preserving nutritional quality. Widespread implementation of nutrition wellness policy provisions could help to narrow the inequities in food access among children in the US. Given the variation in the comprehensiveness of policies, future advocacy and research efforts should focus on the gaps in policy implementation.

Conclusions. The papers in this Special Issue document that the nutritional quality of meals served through the NSLP and SBP, and throughout the school day, have improved significantly over the past decade. These improvements have been across elementary, middle, and high schools and played a major role in reducing disparities in healthy school meals. Yet, there is room for improvement. Competitive foods/beverages contribute to poorer diet quality and added sugars are a major source of calories in children’s diets at school. A limit on added sugars for meals and competitive foods should be incorporated into federal nutrition standards. The findings in this Special Issue emphasize the importance of policies for strengthening nutrition standards and school food practices. Continued action at federal, state, and local levels is needed to ensure all children have access to healthy foods and beverages at school and to reverse upward trends in food insecurity and obesity.

## Data Availability

The data discussed in this paper are restricted-use and come from a large, nationally representative study of the school meal programs that operate in the United States. Requests for access to the public use version of these data should be submitted via electronic mail to FNSStudies@usda.gov.
